# Intracranial Pressure as an Objective Biomarker of Decompression Adequacy in Large Territory Infarction: A Multicenter Observational Study

**DOI:** 10.3389/fsurg.2022.823899

**Published:** 2022-05-06

**Authors:** Jia Xu Lim, Sherry Jiani Liu, Tien Meng Cheong, Seyed Ehsan Saffari, Julian Xinguang Han, Min Wei Chen

**Affiliations:** ^1^ National Neuroscience Institute, Department of Neurosurgery, Singhealth, Singapore, Singapore; ^2^ Center for Qualitative Medicine, Duke-NUS Medical School, National University of Singapore, Singapore, Singapore

**Keywords:** large territory infarctions, decompressive craniectomy, intracranial pressure, modified Rankin scale, outcomes, middle cerebral artery infarction, malignant infarction, hyperosmolar therapy

## Abstract

**Background:**

Decompressive craniectomy (DC) improves the survival and functional outcomes in patients with malignant cerebral infarction. Currently, there are no objective intraoperative markers that indicates adequate decompression. We hypothesise that closure intracranial pressure (ICP) correlates with postoperative outcomes.

**Methods:**

This is a multicentre retrospective review of all 75 DCs performed for malignant cerebral infarction. The patients were divided into inadequate ICP (iICP) and good ICP (gICP) groups based on a suitable ICP threshold determined with tiered receiver operating characteristic and association analysis. Multivariable logistic regression was performed for various postoperative outcomes.

**Results:**

An ICP threshold of 7 mmHg was determined, with 36 patients (48.0%) and 39 patients (52.0%) in the iICP and gICP group, respectively. After adjustment, postoperative osmotherapy usage was more likely in the iICP group (OR 6.32, *p* = 0.003), and when given, was given for a longer median duration (iICP, 4 days; gICP, 1 day, *p* = 0.003). There was no difference in complications amongst both groups. When an ICP threshold of 11 mmHg was applied, there was significant difference in the duration on ventilator (ICP ≥11 mmHg, 3–9 days, ICP <11 mmHg, 3–5 days, *p* = 0.023).

**Conclusion:**

Surgical decompression works complementarily with postoperative medical therapy to manage progressive cerebral edema in malignant cerebral infarctions. This is a retrospective study which showed that closure ICP, a novel objective intraoperative biomarker, is able to guide the adequacy of DC in this condition. Various surgical manoeuvres can be performed to ensure that this surgical aim is accomplished.

## Introduction

Malignant cerebral infarction, occurring in up to 10% of all strokes (1), is defined as a large territory stroke associated with significant progressive cerebral edema, leading to raised intracranial pressure (ICP), cerebral herniation, and subsequently death. Treated medically alone (2–4), mortality is up to 80% (5–7). Multiple randomised controlled trials have shown clear evidence of significant reduction in mortality and some improvement in functional outcomes (8–14) when timely surgical decompression is performed.

The primary surgical goal of decompressive craniectomy (DC) is the control of ICP either prophylactically or therapeutically (2, 15–17). The 2014 scientific statement released by the American Heart Association and American Stroke Association (2) suggested that a bone flap of ≥12 cm is performed in DC. In an attempt to further optimise the procedure, numerous other studies have been conducted including the investigation of different bone flap sizes (18–24), retroauricular surgical incision (25), resection of the temporalis muscle (26), temporal lobectomy (20, 21, 27–29), and various forms of duroplasty (30, 31). These adjunctive manoeuvres, although effective, have significant disadvantages such as the risk of intraoperative or postoperative haemorrhage in lobectomies (20, 21, 27–29) and cosmetic and masticatory defects for temporalis resection (26). Hence, they should only be employed in situations where the standard DC is unable to sufficiently alleviate the raised ICP. In addition, none of these recommendations, account for the varying severity of the underlying pathology (32–35) and differences in an individual’s cranial anatomy.

Judgement of the adequacy of decompression is, however, highly subjective. Although objective methods that compared the size of DC and severity of brain shifts have been employed, this was performed using postoperative computed tomography (CT) imaging (36), which renders immediate intraoperative remedies impossible. Furthermore, raised ICP postoperatively in malignant cerebral infarctions has been shown to correlate with poorer outcomes, both in the short (37) and the long term (38), and has been used as a therapeutic target (39, 40).

We hypothesise that the ICP values on closure is firstly, correlated with postoperative outcomes; secondly, a variable that can be actively corrected; and hence, can be used as an objective intraoperative biomarker to guide surgeons in the determination of the adequacy of the surgical decompression.

## Methods

### Study Design

This multicentre retrospective review was performed in Tan Tock Seng Hospital and Singapore General Hospital, two of the major comprehensive stroke and neurosurgical centres in Singapore, from February 2016 to August 2020. All patients who underwent surgical decompression for cerebral infarctions were recruited. Institutional review board approval was obtained.

### Inclusion and Exclusion Criteria

All adult patients from 18 to 80 years of age who underwent DC for large territory supratentorial cerebral infarction were included. These patients either underwent prophylactic or therapeutic decompression, after developing malignant cerebral edema and deteriorating neurologically. Patients fulfilling the selection criteria of the institutional protocol were considered for surgery. The inclusion criteria were: (1) large territory infarctions defined as an acute cerebral infarction involving more than 50% of the middle cerebral artery territory on neuroimaging, (2) occurring within 96 h from onset of stroke, and (3) a good premorbid functional status of modified Rankin scale (MRS) <3. The exclusion criteria were: (1) having a poor premorbid status of MRS ≥3, (2) poor preoperative neurological status of Glasgow coma scale (GCS) <6 or bilateral mydriasis, (3) severe haemorrhagic transformation involving >30% of infarction zone, and (4) medical comorbidities precluding surgical treatment such as life expectancy <3 years, uncorrected coagulopathy, and severe medical comorbidities. Patients with infratentorial infarctions and infarctions secondary to postoperative complications or trauma were excluded from the analysis.

### Operative Steps and Perioperative Management

The DC was performed with a large reverse question mark incision with a convexity craniectomy of at least 12 cm diameter and subtemporal decompression. A temporal lobectomy may be performed at the surgeon’s discretion, if there was severe intraoperative brain swelling or if the surgical decompression was deemed insufficient. A strain gauge intraparenchymal ICP monitor (Codman® Microsensor® ICP transducer) was then inserted into the ipsilateral middle frontal gyrus and dural substitute was overlaid before closure in layers. The ICP was recorded at the end of closure.

Postoperatively the patients were managed in the neuroscience intensive care unit (NICU) using a tiered protocol with the aim of keeping ICP less than 20 (41, 42). The basic tier includes positional measures (head up and neck neutral), and prevention of physiological and metabolic derangements (fever, seizures, etc). Subsequent tiers include osmotherapy (10% mannitol and/or hypertonic saline), then paralysis, and then barbiturate coma. Once the patient was determined to have adequate postoperative ICP control and was over the period of malignant swelling, the ICP treatment was sequentially weaned off. The patient gets extubated or tracheostomised based on their neurological status and ability to maintain airway.

### Data Collection

Patient characteristics, including age, sex, premorbid clinical status defined with ambulation and the ability to perform activities of daily living (ADL), were recorded. This is in addition to the modified frailty index (MFI-11) (43), an aggregate score that measures patient’s state of frailty based on medical history. Preoperative GCS and pupillary dilatation were noted. Radiological features of the infarction were recorded, including laterality of stroke, presence of haemorrhagic conversion, midline, and brainstem shift, uncal herniation, and of an internal carotid artery infarction. Internal carotid artery infarction was defined as a complete middle cerebral artery infarction with either anterior and/or posterior cerebral artery territory involvement. The surgical intent, prophylactic, or therapeutic, and additional intraoperative manoeuvres such as lobectomy and temporalis resection along with the ICP reading on closure were also detailed.

Inpatient outcomes measured included the length of stay in NICU, usage of osmotherapy (mannitol and/or hypertonic saline) and barbiturates for ICP control, and duration on ventilator. Complications including repeat surgery and syndrome of trephine (44) were noted also. Long term outcomes included mortality at 30 days and 6 months, and MRS at 3 and 6 months. MRS of 0–2 was defined as favourable in our study.

### Statistical Analysis

Patients were grouped into inadequate ICP (iICP) or good ICP groups (gICP) based on an ICP threshold derived using tiered receiver operating characteristic (ROC) and association analysis. These processes were described in detail within the supplementary notes. Model performance indices including the beta coefficient, the area under the ROC curve (AUC), sensitivity and specificity, along with their 95% CI were reported.

Categorical variables were described using frequency (%); and continuous variables were reported as mean ± standard deviation or median (IQR). Baseline characteristics were compared using Chi square test (or Fisher exact test, where appropriate) and two-sample t-test (or Mann-Whitney U test, depending on the normality assumption) for categorical and continuous variables, respectively. Multivariable logistic regression was performed to investigate outcomes after adjusting for age, preoperative GCS, and surgical intent of DC. Un-adjusted and adjusted odds ratios (OR) and 95% confidence intervals (CI) were reported. Firth’s penalized likelihood approach was applied for rare events in logistic regression analysis. Data analysis was performed using SAS software version 9.4 for Windows (Cary, NC: SAS Institute Inc.) and statistical significance was set at *p* < 0.05.

## Results

### Patient Characteristics

A total of 81 DCs were performed during the study period. After application of exclusion criteria, 75 DCs were included in the final analysis. An ICP threshold of 7 mmHg (<7 vs ≥7 mmHg) was identified when examining ventilator days, and postoperative osmotherapy for ICP. There were 36 patients (48.0%) assigned into the iICP and 39 patients (52.0%) into the gICP group.

**Figure 1:**
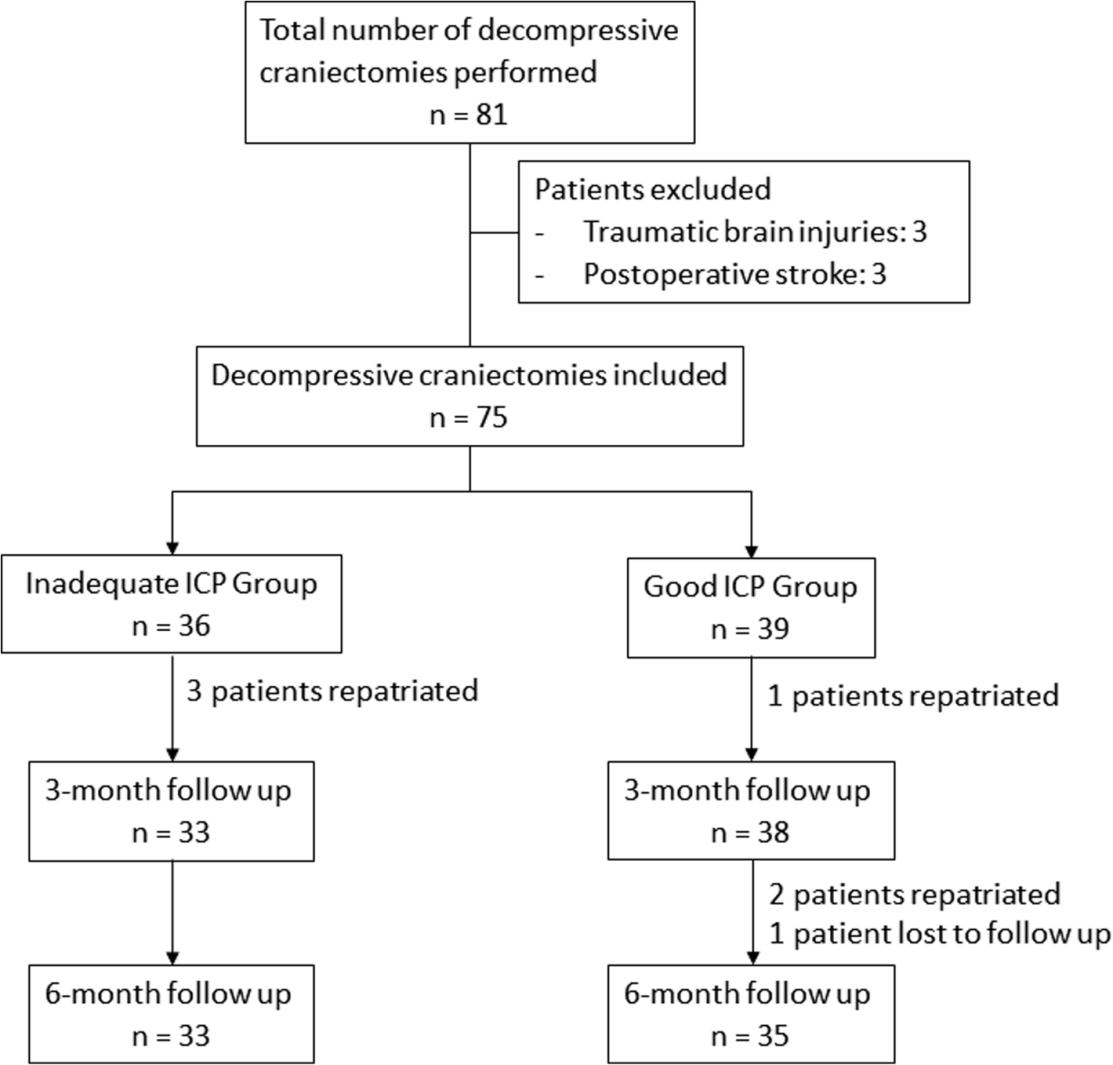
Flowchart of patients at various timepoints. Outcome evaluation of decompression at 3 and 6-month follow up.

Both groups were comparable in terms of mean age (iICP, 54.6 ± 11.9 years; gICP, 58.3 ± 11.4 years), sex (iICP, 75.0% males; gICP, 61.5% males), premorbid ADL status (iICP, 100% independent; gICP, 100%), preoperative median GCS (iICP, 9; gICP, 10), and the presence of anisocoria (iICP, 36.1%; gICP, 33.3%). Radiological features, surgical intent and intraoperative performance of lobectomy were likewise similar. Of note, the median MRS was significantly better in the iICP group (*p* = 0.018). No patients in our cohort had temporalis resection performed during DC. These details were summarised in Table 1. Figure 1 demonstrates the number of patients included and remaining at each timepoint.

**Table 1:** Patient characteristics amongst both groups using ICP threshold of 7 mmHg.

		Inadequate ICP (*n* = 36)	Good ICP (*n* = 39)	OR (95% CI)	*P* value
Age	Mean ± SD	54.6 ± 11.9	58.3 ± 11.4	–	0.17
	Range	24–75	29–77	–	
Male Gender	Frequency (%)	27 (75.0)	24 (61.5)	1.88 (0.70–5.06)	0.21
Independent ADL	Frequency (%)	36 (100)	37 (100)	–	1.00
Community Ambulant	Frequency (%)	36 (100)	37 (94.9)	–	0.39
MRS	Median (1Q – 3Q)	0 (0–0)	0 (0–0)	–	**0** **.** **018**
MFI-11	Median (1Q – 3Q)	0.5 (0–1)	1 (0–2)	–	0.068
Preoperative GCS	Median (1Q – 3Q)	9 (7.25–12.5)	10 (8–11)	–	0.63
Anisocoria	Frequency (%)	13 (36.1)	13 (33.3)	1.13 (0.44–2.93)	0.80
Stroke Characteristic					
Left sided	Frequency (%)	18 (50.0)	20 (51.3)	0.95 (0.38–2.35)	0.91
Haemorrhagic conversion	Frequency (%)	23 (63.9)	28 (71.8)	0.70 (0.26–1.84)	0.46
Midline shift present	Frequency (%)	31 (86.1)	31 (79.5)	1.6 (0.47–5.44)	0.55
Midline Shift (mm)	Mean ± SD	7.9 ± 5.9	7.5 ± 6.0	–	0.75
Brainstem Shift	Frequency (%)	10 (27.8)	15 (38.5)	0.62 (0.23–1.63)	0.33
Uncal herniation	Frequency (%)	18 (50.0)	24 (61.5)	0.63 (0.25–1.57)	0.32
ICA Infarction	Frequency (%)	12 (33.3)	20 (51.3)	0.48 (0.19–1.21)	0.12
Therapeutic Surgical Intent	Frequency (%)	20 (55.6)	24 (61.5)	0.78 (0.31–1.96)	0.60
Lobectomy	Frequency (%)	5 (13.9)	4 (10.3)	1.41 (0.35–5.73)	0.73

*ICP, intracranial pressure; ADL, activities of daily living; MRS, modified Rankin scale; MFI, modified frailty index; GCS, Glasgow coma scale; ICA, internal carotid artery.*

### Inpatient and Long-Term Outcomes

In terms of postoperative management, the iICP group were more likely to require osmotherapy (iICP, 83.3%; gICP, 51.4%, *p* = 0.003) and when given, required it for a longer median duration (iICP, 4 days, gICP, 1 day, *p* = 0.003). Otherwise, barbiturate use and duration, days on ventilator and length of stay in NICU were statistically similar amongst both groups.

Significantly, there were no differences in the rates of repeat surgery (iICP, 2.8%; gICP, 0%), syndrome of the trephine (iICP, 2.8%; gICP, 2.6%), median discharge GCS (iICP, 14; gICP, 13.5) and inpatient mortality (iICP, 22.2%; gICP 17.9%) amongst both groups.

When considering outcomes after discharge, the proportion of patients with 30-day mortality (iICP, 22.9%; gICP, 17.9%), 6-month mortality (iICP, 26.5%; gICP, 22.9%), 3-month favourable MRS (iICP, 6.1%; gICP, 0%) and 6-month favourable MRS (iICP, 15.2%; gICP 8.6%) were alike. The above details are described in Table 2.

**Table 2:** Outcomes using ICP threshold of 7 mmHg.

			Inadequate ICP (*n* = 36)	Good ICP (*n* = 39)	Unadjusted		Adjusted	
					OR (95% CI)	*P* Value	OR (95% CI)	*P* Value
Osmotherapy	Given	Frequency (%)	30 (83.3)	19 (51.4)	4.74 (1.60–14.0)	0.004	6.32 (1.88–21.2)	0.003
	Duration	Median (1Q – 3Q)	4 (1.25–5)	1 (0–4)	–	0.009	–	0.003
Barbiturate	Given	Frequency (%)	5 (13.9)	2 (5.4%)	2.82 (0.51–15.6)	0.26	3.30 (0.51–21.5)	0.21
	Duration	Median (1Q – 3Q)	0 (0–0)	0 (0–0)	**–**	0.20	–	0.12
Days on Ventilator		Median (1Q – 3Q)	4 (3–7.75)	3 (3–5)	–	0.28	–	0.14
NICU Length of Stay		Median (1Q – 3Q)	5.5 (4–10)	5 (4–8)	–	0.46	–	0.27
Repeat Surgery		Frequency (%)	1 (2.8)	0 (0)	–	0.48	–	1.00
Syndrome of Trephine		Frequency (%)	1 (2.8)	1 (2.6)	1.09 [0.07–18.0]	1.0	1.24 (0.67–23.0)	0.89
Tracheostomy		Frequency (%)	2 (5.6)	2 (5.1)	1.09 [0.15–8.16]	1.0	1.15 (0.15–9.00)	0.89
Discharge GCS		Median (1Q – 3Q)	14 (11–15)	13.5 (11–15)		0.85	–	0.55
Mortality	Inpatient	Frequency (%)	8 (22.2)	7 (17.9)	1.31 [0.42–4.06]	0.64	1.70 (0.48–6.05)	0.42
	30 day	Frequency (%)	8 (22.9)	7 (17.9)	1.35 (0.44–4.22)	0.60	1.62 (0.45–5.91)	0.46
	6 months	Frequency (%)	9 (26.5)	8 (22.9)	1.22 (0.41–3.64)	0.73	1.50 (0.41–5.45)	0.54
MRS	Favourable MRS at 3m	Frequency (%)	2 (6.1)	0 (0)	–	0.21	–	1.00
	Favourable MRS at 6m	Frequency (%)	5 (15.2)	3 (8.6)	1.91 (0.42–8.70)	0.47	1.68 (0.30–9.31)	0.56

*ICP, intracranial pressure; NICU, neuroscience intensive care unit; GCS, Glasgow coma scale; MMRS, modified Rankin scale.*

For the duration of ventilator usage and length of stay in NICU, an ICP threshold of 11 mmHg differentiated our cohort. The group of patients with ICP of ≥11 mmHg had a significantly longer median duration on mechanical ventilation (ICP ≥11 mmHg, 4 [3–9] days; ICP <11 mmHg, 4 [3–5] days, *p* = 0.023) and a trend towards a longer stay in the NICU (ICP ≥11 mmHg, 6 [5–10] days; ICP <11 mmHg, 5 [4–8] days, *p* = 0.065). This information was documented in Table 3.

**Table 3:** Adjusted ventilator days and length of stay in NICU using ICP threshold of 11 mmHg.

		Inadequate ICP (*n* = 15)	Good ICP (*n* = 60)	Beta (95% CI)	*P* value
Days on Ventilator	Median (1Q – 3Q)	4 (3–9)	4 (3–5)	0.43 (0.06–0.81)	0.023
Length of Stay in NICU	Median (1Q – 3Q)	6 (5–10)	5 (4–8)	0.30 (−0.02–0.62)	0.065

*ICP, intracranial pressure; NICU, neuroscience intensive care unit.*

## Discussion

DC has been shown to reduce mortality effectively in malignant cerebral infarctions (8–14). However, there is no known objective gauge that indicates if the surgical aim of sufficient ICP control were met. Our study demonstrated that the novel usage of the closure ICP was correlated with the extent of postoperative ICP medical management and hence can be used as an objective biomarker to indicate adequate decompression.

### Measurement of Intraoperative and Postoperative ICP

The central hypothesis behind this study hinges upon the immediate and accurate measurement of ICP on closure. Due to this requirement, a strain gauge ICP monitor is used. Various studies have demonstrated that the Codman ICP monitor is consistent and precise even when compared against the ICP measured from an external ventricular drain (EVD) (45–48). In the authors’ opinion, intraoperative ICP measurements using other methods such as fibre-optic or a fluid-filled catheter, may not be able to achieve accurate measurements promptly and hence are not suitable for the intraoperative determination of decompression adequacy.

### Postoperative ICP Control

ICP control is an interplay between surgical decompression and postoperative medical therapy. A closure ICP ≥7 mmHg was shown to be associated with difficult postoperative ICP control, and the closure ICP ≥11 mmHg was related to an increased duration of mechanical ventilation. Although osmotherapy usage was significantly different amongst both groups, barbiturate usage was likely not significant due to the small numbers that required it (9.3% of cohort).

Malignant cerebral infarction is known to have progressive swelling (49, 50). This delayed phenomenon occurs when the previously at-risk penumbral tissue progresses to infarction followed by delayed swelling, and in some cases haemorrhagic transformations (29). Hence, despite both thresholds of 7 and 11 mmHg are traditionally considered “low-normal”, if not attained, the likelihood of the progressive swelling leading to difficulties in controlling ICP at its peak, around day two to five after stroke (49, 50), is substantial. Furthermore, there is some evidence that the postoperative target for malignant cerebral infarctions should be lower than the 20 mmHg commonly used in traumatic brain injuries (37, 51, 52), further lending credence to the notion that a lower ICP target should be the goal. In addition, the similar rates of re-operation and syndrome of the trephine amongst both groups demonstrates that further decompression to “low-normal” ICP targets is safe and well tolerated by patients.

One unexpected outcome was the similar duration of mechanical ventilation and length of NICU stay despite a reduced need for medical treatment for ICP treatment in the gICP group. Mechanical ventilation, and hence NICU stay, is necessary when patients are placed on deep sedation on top of osmotherapy. One reason for this could be related to undocumented non-ICP reasons that require prolonged intubation such as pneumonia (53), delayed recovery in GCS, or a small sample size that is not able to detect a difference.

Significantly, despite having a lower closure ICP, iICP and gICP patients had similar inpatient and long-term outcome. This was likely due to the postoperative medical treatment making up for the varying degrees of adequacy of surgical decompression. This was corroborated by the similarly low rates of re-operation for iICP group.

### Intraoperative Decision Making During Surgical Decompression

After the completion of a standard DC, the closure ICP should be estimated by bringing the skin flap to the opposite skin edge. Should the ICP value at this point be ≥7  mmHg, the authors recommend the sequential performance of the following manoeuvres: DC extension, temporal lobectomy, temporalis resection, and EVD insertion. The order of performance was suggested based on the surgical difficulty and risk involved. The first option, DC extension, is safe and easily executed in the author’s experience. After dissecting the dura off the bone edge under direct vision, a 1–2 cm rim of bone is then removed. This mitigates the risk of breaching the dural venous sinus. At this point, the closure ICP should be reassessed and should it remain inadequate, the performance of further manoeuvres should be considered. Temporal lobectomy and EVD insertion have an elevated risk of haemorrhage, especially since there will often be antithrombotics given at some stage after presentation of the stroke, while temporalis resection, although effective, leads to cosmetic deficiencies and masticatory dysfunction (26).

### Surgical Decompression as Part of Overall Stroke Management

From prehospital management by emergency medical services (54), establishment of stroke centres (55) and mobile stroke units (56), to advances in stroke imaging (57, 58) and extension of the thrombolysis (59) and thrombectomy window (60, 61), stroke management has advanced significantly in the past decades. In light of these developments, surgical decompression, retains its role as the last resort in “end stage” cerebral infarction. When performed well, with clear and objective intraoperative surgical targets to achieve, DC has the potential to influence ICP control postoperatively and potentially reduce the need for ventilation and NICU. This may contribute to further improvements in overall stroke outcomes along with significant cost reductions during intensive care. Economic analysis of the effect of an adequate surgical decompression were beyond the scope of our study and hence was not further explored. We recommend that further large-scale prospective studies and economic analysis can be conducted to further validate this claim.

### Limitations

This study suffers from the inherent limitations of a retrospective review. It was, however, mitigated with the use of multivariable logistic regression analysis. Another limitation of our study was the number of patients lost to follow up (7 patients [9.3%]). This effect was minimised as both groups had a similar proportion of such patients.

## Conclusion

We established that patients with a closure ICP of <7 mmHg had a lesser need for prolonged postoperative medical management and closure ICP <11 mmHg was associated with a reduced duration on mechanical ventilation and reduced length of NICU stay. This is a retrospective study which showed that closure ICP, a novel objective intraoperative biomarker, is able to guide the adequacy of DC in malignant cerebral infarctions. Various surgical manoeuvres can be performed to ensure that this surgical aim is accomplished.

## Data Availability

The raw data is available at reasonable request to the corresponding author.
