# Supplementary material for: Intracranial Pressure as an Objective Biomarker of Decompression Adequacy in Large Territory Infarction: A Multicenter Observational Study
Source: Front Surg. 2022 May 6;9:823899. doi: 10.3389/fsurg.2022.823899 (PMC9235838; doi:10.3389/fsurg.2022.823899)
Supplement: Supplementary file 1 [file fsurg-09-823899_Data_Sheet1_v1.pdf]

Supplementary Material

ICP as an objective marker of decompression adequacy in malignant cerebral infarction: a multicentre observational study

Supplemental Methods

Outcome variables were ranked into tiers during consideration for intracranial pressure (ICP) threshold. These includes, in the first tier: long term functional outcome (modified Rankin scale [MRS]) and mortality; second tier: duration of mechanical ventilation and length of stay (LOS) in the neuroscience intensive care unit (NICU); and the third tier: usage of various forms of osmolar therapy and barbiturates as a surrogate for postoperative ICP control. Each tier is measured against closure ICP using either receiver operating characteristic (ROC) analysis or negative binomial regression analysis (for binary outcome and count outcome variables, respectively) and if no meaningful model performance can be derived, the next tier is interrogated sequentially.

In our study, the first-tier outcomes of favourable MRS and mortality at 6 months were both unremarkable with an area under the ROC curve (AUC) of 0.57 and 0.60, respectively. The second-tier outcomes of mechanical ventilation duration and NICU LOS had a significant ICP threshold of 11 mmHg (<11 vs ≥11 mmHg). The group with ICP ≥11 mmHg was associated with a 0.43 (95% CI 0.06 – 0.81) higher expected logarithm of duration on ventilation ($p = 0.023$) and 0.30 (95% CI 0.02 – 0.62) longer expected logarithm of NICU LOS ($p = 0.065$). Of note, ICP ≥7 mmHg trended towards significance when looking at duration on mechanical ventilation (Beta 0.30 [95% CI -0.001 – 0.61], $p = 0.058$). When interrogating the third tier, the ICP threshold of 7 mmHg (<7 vs ≥7 mmHg) was significant for duration of osmolar therapy and trended towards significance for duration of ventilation. The group with ICP ≥7 mmHg was associated significantly with a 0.56 (95% CI 0.12 – 1.00) higher expected logarithm of duration of mannitol use ($p = 0.013$) and 1.64 (95% CI 0.19 – 3.09) higher expected logarithm of duration of hypertonic saline use ($p = 0.027$). These information were summarised in Supplemental Tables I and II.

With the above ROC and negative binomial regression analysis results, two ICP thresholds of 7 mmHg and 11 mmHg were found to be suitable. An ICP of 7 mmHg was selected as the overall threshold as 1) the lower ICP threshold of 7 mmHg encompasses an ICP of 11 mmHg; and 2) this threshold is correlated to more outcome variables.

The sample population was divided accordingly to the inadequate ICP group (ICP ≥7 mmHg) and good ICP group (ICP <7 mmHg), with 36 and 39 patients, respectively.

Supplemental Tables

Outcome Variable	Beta* (95% CI)	P Value	Odds Ratio** (95% CI)	P Value
Duration of Ventilation	0.43 (0.06 – 0.81)	**0.023**	-	-
NICU LOS	0.30 (-0.02 – 0.62)	**0.065**	-	-
Duration of Mannitol Usage	0.42 (0.12 – 0.96)	0.13	-	-
Duration of Hypertonic Saline Usage	1.22 (-0.40 – 2.83)	0.14	-	-
Duration of Barbiturate Usage	1.00 (-1.27 – 3.26)	0.39	-	-
Mannitol Given	-	-	2.28 (0.51 – 10.2)	0.28
Hypertonic Saline Given	-	-	4.7 (1.30 – 17.0)	**0.019**
Barbiturate Given	-	-	3.39 (0.71 – 16.2)	0.13

Table I: Outcomes using the ICP threshold of 11 mmHg

An ICP threshold of 11 mmHg is significantly associated with the duration of mechanical ventilation and trend towards significance in the length of stay in NICU.

* Beta coefficients are calculated using univariate negative binomial regression models for the count outcome variables

** Odds ratios are calculated using univariate logistic regression models for the binary outcome variables

Outcome Variable	Beta* (95% CI)	P Value	Odds Ratio** (95% CI)	P Value
Duration of Ventilation	0.30 (-0.001 – 0.61)	0.058	-	-
Duration of Mannitol Usage	0.56 (0.12 – 1.001)	**0.013**	-	-
Duration of Hypertonic Saline Usage	1.64 (0.19 – 3.09)	**0.027**	-	-
Duration of Barbiturate Usage	1.57 (-0.40 – 3.54)	0.12	-	-
Mannitol Given	-	-	2.59 (0.87 – 7.70)	0.088
Hypertonic Saline Given	-	-	3.91 (1.03 – 14.8)	**0.045**
Barbiturate Given	-	-	2.48 (0.51 – 12.2)	0.26

<u>Table II: Outcomes using the ICP threshold of 7 mmHg</u>

An ICP threshold of 7 mmHg is significantly associated with shorter duration of usage of both mannitol and hypertonic saline and has a reduced need for hypertonic saline usage. There is also a trend towards significance in the duration of mechanical ventilation with this threshold.

* Beta coefficients are calculated using univariate negative binomial regression models for the count outcome variables

** Odds ratios are calculated using univariate logistic regression models for the binary outcome variables
